# A global urban microwave backscatter time series data set for 1993–2020 using ERS, QuikSCAT, and ASCAT data

**DOI:** 10.1038/s41597-022-01193-w

**Published:** 2022-03-16

**Authors:** Steve Frolking, Tom Milliman, Richa Mahtta, Aaron Paget, David G. Long, Karen C. Seto

**Affiliations:** 1 Institute for the Study of Earth, Oceans, and Space, University of New Hampshire, 8 College Rd., Durham, NH 03824 USA; 2 Yale School of the Environment, 195 Prospect Street, Yale University, New Haven, CT 06511 USA; 3 Department of Physical and Environmental Sciences, Concord University, Vermillion Street PO Box 1000, Athens, WV 24712 USA; 4 Electrical and Computer Engineering Department, 450 Engineering Building, Brigham Young University, Provo, UT 84602 USA

**Keywords:** Geography, Energy supply and demand, Environmental impact

## Abstract

Urban settlements are rapidly growing outward and upward, with consequences for resource use, greenhouse gas emissions, and ecosystem and public health, but rates of change are uneven around the world. Understanding trajectories and predicting consequences of global urban expansion requires quantifying rates of change with consistent, well-calibrated data. Microwave backscatter data provides important information on upward urban growth – essentially the vertical built-up area. We developed a multi-sensor, multi-decadal, gridded (0.05° lat/lon) data set of global urban microwave backscatter, 1993–2020. Comparison of backscatter from two C-band sensors (ERS and ASCAT) and one Ku-band sensor (QuikSCAT) are made at four invariant non-urban sites (~3500 km^2^) to evaluate instrument stability and multi-decadal pattern. For urban areas, there was a strong linear correlation (overall R^2^ = 0.69) between 2015 ASCAT urban backscatter and a continental-scale gridded product of building volume, across 8450 urban grid cells (0.05° × 0.05°) in Europe, China, and the USA. This urban backscatter data set provides a time series characterizing global urban change over the past three decades.

## Abstract (summary)

**Table:** 

Measurement(s)	land surface microwave backscatter
Technology Type(s)	space-borne scatterometers
Factor Type(s)	urban land
Sample Characteristic - Environment	urban land
Sample Characteristic - Location	global

## Background & Summary

Urban population has risen from 1.0 billion in 1960 (34% of total global population), to 2.3 billion in 1990 (43%), to 4.4 billion in 2020 (56%)^1^. Along with this rapid growth in population, there has been extensive development of urban built-up land and infrastructure^2^.

Space-borne remote sensing provides important datasets for global urban change analysis due to global coverage with uniform reporting (unlike statistical data), availability of multi-decadal time series, and multiple sensors capturing different aspects of global urban change. For example, optical/NIR sensors (e.g., MODIS, Landsat) are used for land classification and urban lateral spread^3–5^; thermal IR sensors (e.g., MODIS, Landsat, GOES) can detect urban heat islands^6^; night-time light sensors (e.g., DMSP, VIIRS) have been used to map impervious surfaces and economic activity^7–9^; lidar can map building structure^10^ but there is no space-borne, full-coverage global product; and SAR microwave sensors can map building structural details^11–13^ but neither global nor multi-decadal records focused on urban land have been assembled. Microwave scatterometer data have the longest time series of global radar backscatter data, spanning from 1978 (SeaSat) to the present (e.g., Advanced Scatterometer or ASCAT). Several studies using data from SeaWinds on QuikSCAT (hereafter QuikSCAT) (1999–2009) have shown that microwave backscatter contains useful information on urban built structure^14–16^, urban lateral spread^17^, and local to global urban growth and development^2,18,19^. Our goal with this data set is to provide an accessible and comprehensive microwave remote sensing record of urban backscatter over the period 1993–2020 in quasi-decadal blocks by sensor: 1993–2000 from the ERS scatterometer; 1999–2009 from QuikSCAT, and 2007–2020 from ASCAT.

All high temporal frequency space-borne remote sensing data have short-term variability (daily to seasonal) due to a variety of factors. Microwave backscatter is highly sensitive to surface roughness and to dielectric properties, which for land can be dominated by liquid water content^20^. Scatterometer data have seasonal variability related to vegetation phenology, even for urban areas^15^, and to landscape freeze-thaw^21–23^. Urban backscatter is dominated by strong corner reflections associated with built structure and ground geometry^14,20^, and large cities with manifold buildings generally have higher backscatter than natural and agricultural areas in both 13.6 GHz Ku-band^14^ and 5.3 GHz C-band^24^, with the strongest backscatter generally coming from the most densely built-up areas^14,16–18^. At a scale of ~10 km, satellite-based sensing of urban backscatter is dependent on the regularity of a city’s street network and the road grid orientation relative to the microwave sensor^15^. The strength of this azimuthal effect varies by city, but it was shown to be fairly constant over 1999–2009, including cities where the total urban backscatter strength over that decade showed a strong trend^15^. Based on these considerations as well as the overall goal of developing a metric relevant for urban change, we have developed time series of urban monthly and seasonal mean backscatter, from which an annual time series can be constructed with a goal of avoiding freeze/thaw, averaging over high-frequency variability due to weather, and using an interval with fairly constant vegetation backscatter.

**Fig. 1:**
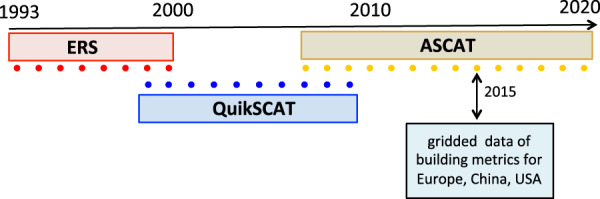
Schematic overview of the urban backscatter data sets. Shaded bars represent range of sensor data (see Methods) and solid circles represent summer season means for each year for ERS (1993–2000), QuikSCAT (1999–2009), ASCAT (2007–2020). Urban data domain masks: 0.05° lat/lon gridded backscatter data were masked by open water minimum threshold of 50%, and GHSL (Global Human Settlement Layer) 2014 built-fraction minimum threshold of 20%. ASCAT 2015 urban backscatter is compared to gridded building volume and height data for Europe, China, and the USA.

Here we construct quasi-decadal, temporally overlapping, monthly and seasonal urban backscatter time series using microwave scatterometer data from ERS (1/1993 – 12/2000), QuikSCAT (07/1999–11/2009), and ASCAT (1/2007–12/2020) sensors (Fig. 1). We then mask these products to global urban areas, and compare these urban backscatter data to a continental-scale building metrics data set for >200 large cities. Finally, we generate some sample urban backscatter time series to illustrate the product. Numerous long-term datasets are available to study the time series of outward urban expansion. However, continuous time series datasets to understand the upward urban expansion are not available. Our aim here is to fill this gap by constructing a multi decadal time series dataset which will help in understanding long-term upward urban growth.

## Methods

### Microwave backscatter data

We selected images from three backscatter data collections with quasi-decadal data time series that have been processed by and are available from the NASA Scatterometer Climate Record Pathfinder (SCP) project (https://www.scp.byu.edu/). These collections are: ERS-1/2^25^, SeaWinds on QuikSCAT^26^, and Enhanced Resolution ASCAT^27^ (Table 1). QuikSCAT is a Ku-band scatterometer (13.6 GHz) with rotating inner (HH polarization) and outer (VV polarization) pencil beam antennas, while ERS-1/2 and ASCAT-A/B are C-band scatterometers (5.3 GHz) with fixed swath VV antennas. C- and Ku-band microwaves have inherently different backscatter strengths from most land surfaces^20^. Calibration of the ASCAT sensors is good enough that multiple ASCAT instruments can be treated as a single sensor. ERS-1/2 are similarly well calibrated against each other and we combine these into a single time series.

**Table 1:** Backscatter sensor image data included in this dataset.

Sensor	Freq. (Ghz)	Image class	Polarization	Nominal pixel resolution (km)	Effective sensor resolution (km)	Image frequency	Image era
ERS-1	5.3	ers1	VV	8.9	35 [ref. ^42^]	overlapping 17-day	1993–1995*
ERS-2	5.3	ers2	VV	8.9	35 [ref. ^42^]	overlapping 17-day	1996–2000
QuikSCAT	13.4	quev	VV	4.45	11 [ref. ^29^]	non-overlap 4-day	1999–2009
ASCAT	5.3	msfa	VV	4.45	15-20 [ref. ^43^]	overlapping 5-day	2007–2020

(Data source: https://www.scp.byu.edu/). *ERS-1 data from 1992 had substantial gaps and striping, and were excluded from the dataset.

The backscatter images are multi-day composites made from the original L1B data for individual passes, and are created using the Scatterometer Image Reconstruction (SIR) algorithm^28,29^. The image data are separated by geographical region, each with it’s own Lambert Azimuthal Equal Area (LAEA) projection. The following regions were used: Alaska (Ala); Australia (Aus); Bering Sea (Ber); Central America (CAm); China/Japan (ChJ); Europe (Eur); Greenland (Grn); Indonesia (Ind); North Africa (NAf); North America (NAm); South Africa (SAf); South America (SAm); South Asia (SAs); and Siberia (Sib).

As discussed in Long (2017)^29^, the SIR algorithm provides higher effective resolution than simple gridding algorithms by exploiting the overlapping spatial response function of the measurements to reconstruct the backscatter on a fine resolution grid. The grid size is smaller than the effective resolution (Table 1) to ensure that Nyquist sampling requirements are met. Like all imaging techniques, the spatial response function of the image pixels extends somewhat beyond the pixel size, but falls to negligible values within one or two pixels. We note that the reconstruction acts as a high pass filter to enhance fine details and sharpens image edge. This can lead to Gibbs phenomena overshoot at edges^29^. Note that the re-projection and resampling for this data set reduces these negative effects. The images are available from the BYU site in ‘.sir’ format, and we downloaded the “a” (σ°), as well as the associated land mask images for each region. QuikSCAT VV polarization data were chosen to be consistent with VV polarization C-band data. Note that the SCP “a” values for ERS and ASCAT are σ° at 40° incidence angle computed as σ°(dB) = a + b·(incidence_angle – 40°). The images were converted to geotiff format (with data type = Float) and during the conversion to geotiff format the land mask for each region was applied.

The geotiffs generated from the multi-day .sir images (Table 1) were temporally aggregated for all three sensors both to monthly and to quarterly or “seasonal” (Jan-Mar, Apr-Jun, Jul-Sep, Oct-Dec) mean and standard deviation values. These seasonal values for σ° (in dB) were generated for the entire record for each instrument and for each region.

The data were assembled at 0.05° lat/lon spatial resolution, both for ease of use by a diverse community of researchers, and to be roughly consistent with the spatial resolution of the backscatter data processed and posted by the NASA SCP. We used the GDAL utility, gdalwarp, with the “average” resampling method to reproject the regional images to a lat-lon grid (EPSG:4326) then mosaicked the regions using the gdal_merge.py utility. This procedure results in a near global (58°S to 64.5°N) single image for each month or season. These images were assembled into NetCDF format files for each instrument with seasonal mean, seasonal standard deviation for both urban masked and unmasked (all land) data, and monthly mean and standard deviation for unmasked data.

A full analysis of the uncertainties associated with the backscatter measurements has already been provided in Long (2017)^29^. Extensive information and resources for understanding the uncertainties associated with the image reconstruction are available on the NASA SCP site (https://www.scp.byu.edu/). The standard deviation values that are part of this dataset are an estimate of the variability of the temporal aggregations to generate the monthly and seasonal values.

### Additional data to constrain urban spatial domain

To mask the backscatter data to urban land, the GHSL built fraction (BF) in epoch 2014^30^ was used to identify urban grid cells at 0.05° × 0.05°. We used the 1-km GHSL product (https://ghsl.jrc.ec.europa.eu/ghs_bu2019.php), which was developed from Landsat data^31,32^. The global gridded built fraction data (GHS-BUILT R2018A) were aggregated to 0.05° using the GDAL utility, gdalwarp, using the ‘average’ resampling method, equivalent to what was used with the backscatter data. A minimum built fraction threshold of 20% in 2014 was used to classify urban grid cells, following earlier work by Mahtta *et al*.^2^ and consistent with a comparison of ASCAT and Sentinel-1 SAR data for Nanjing, China^33^. This 2014 built-fraction threshold was applied to all sensors and years, i.e., 1993–2020. Using the most recent (2014) urban cover data from GHSL maximizes the area classified as urban, including land urbanized in the 1990s and 2000s. We explore the sensitivity of this threshold in the figures below by also using a 10% built fraction threshold.

Because *σ*° values are very sensitive to water surfaces, and because many urban areas are located near water bodies, for the urban-masked data we applied an additional mask based on the water fraction in each grid cell. Grid cell water fraction was extracted from the Global 1-km Consensus Land Cover dataset^34^ available at http://www.earthenv.org//landcover. This was aggregated to 0.05° using the same method as for the urban built fraction, and for the urban data set, all 0.05° grid cells with water area >50% were excluded. Backscatter variance increased with increasing grid cell water fraction (e.g., coastal grid cells), and inspection of Shanghai, China at the mouth of the Yangtze River was used to select a threshold of 50%.

The combination of these two masks reduced our dataset from approximately 5.2 million grid cells (land) to 38,525 (urban) 0.05° × 0.05° grid cells. Note that the posted data also include unmasked (land) monthly and seasonal backscatter data sets, so that users can apply other masks to the data.

## Data Records

The data set comprises eighteen NetCDF files at 0.05° (lat/lon) grid resolution. Six files are monthly mean backscatter time series, and backscatter standard deviation time series, for three sensors and eras: ERS (Jan 1993 – Dec 2000), QuikSCAT (Jul 1999 – Nov 2009), and ASCAT (Jan 2007 – Dec 2020) for all land. Twelve files are seasonal mean backscatter time series, and backscatter standard deviation time series, for three sensors and eras: ERS (Jan-Mar/1993 – Oct-Dec/2000), QuikSCAT (Jan-Mar/1999 – Oct-Dec/2009), and ASCAT (Jan-Mar/2007 – Oct-Dec/2020), all for two coverages – urban and all land. The four seasons are January-March, April-June, July-September, and October-December. The urban data files include backscatter σ° values in dB for all four seasons for a subset of all land grid cells between 64.5°N and 58°S, with grid cells masked (no-data value) if they have >50% water cover or GHSL 2014 Built Fraction <20% (i.e., BF_2014_ < 0.2). The land data files include backscatter σ° values in dB for all four seasons or all twelve months for all land grid cells between 64.5°N and 58°S, with only a global land mask. (See *Usage Notes* section below for some additional spatio-temporal data limitations.) Each coverage (land-monthly, land-seasonal, and urban-seasonal) consists of six NetCDF files: mean seasonal backscatter, with one NetCDF file for each of the three sensors, and standard deviation in mean seasonal backscatter, with one NetCDF file for each of the three sensors.

**DATSET NAME**: Global Monthly and Seasonal Urban and Land Backscatter Time Series (1993–2020)

**File format**: NetCDF v.4 with default internal compression (level 7)

**File naming convention**: {*sensor*}_{timestep}_{*coverage*}_sig0_{*variable*}.nc, where {*sensor*} is one of: ‘ERS’ (years 1993–2000), ‘QuikSCAT’ (years 1999–2009), or ‘ASCAT’ (years 2007–2020); {*timestep*} is one of ‘monthly’ or ‘seasonal’; {*coverage*} is one of: ‘urban’ or ‘land’; and {*variable*} is one of: ‘mean’ or ‘StdDev’.

**Date Produced:** July 2021

**Spatial Metadata**:

Extent X: −180 to +180

Extent Y: −58 to +64.5

Extent Z: ERS-seasonal: 32, one layer per season for Jan-Mar/1993 – Oct-Dec/2000.

QuikSCAT-seasonal: 44, one layer per season for Jan-Mar/1999 – Oct-Dec/2009.

ASCAT-seasonal: 56, one layer per season for Jan-Mar/2007 – Oct-Dec/2020.

ERS-monthly: 96, one layer per month for Jan/1993 – Dec/2000.

QuikSCAT-monthly: 125, one layer per month for Jul/1999 – Nov/2009.

ASCAT-monthly: 168, one layer per month for Jan/2007 – Dec/2020.

Resolution: 0.05 decimal degrees

Coordinate reference system: longitude/latitude (WGS84 datum)

Projection in PROJ.4 notation: “+proj = longlat + datum = WGS84 + no_defs”

Temporal resolution: monthly (12/yr) or seasonal (4/yr) Jan-Mar, Apr-Jun, Jul-Sep, Oct-Dec; also see Usage Notes below.

**Units**: backscatter *σ*° in dB.

**No Data value:** −9999. For all grid cells excluded by the NASA SCP land mask, and for high latitudes (>64.5° or <−58° latitude); for the urban coverage, no data values are assigned to grid cells with open-water >50% of grid cell area, or with GHSL built-fraction in 2014 < 20%; also see Methods above, and Usage Notes below.

**Repository:** Socioeconomic Data and Applications Center (SEDAC)^35^; 10.7927/gr2e-dh86. Note that SEDAC requires user registration and login to access data. 

## Technical Validation

In our analyses below, we used the backscatter power return ratio, PR = 10^(σ°/10), where σ° is the backscatter in dB, as reported in the datasets. Backscatter PR had a stronger linear correlation with independent building volume and height data than did σ° (see *Summer ASCAT Backscatter Compared to Building Data* below). For application to urbanization trends, it is important to minimize variability introduced by transient events (i.e., not related to the image reconstruction) that affect backscatter returns, such as weather (precipitation, floods, and fall/winter/spring freeze-thaw signals), and substantial variation in vegetation canopy reflectance. For this reason, we constructed an annual time series using three-month seasonal mean backscatter values during the growing season, i.e., Jul-Sep in the northern hemisphere, and Jan-Mar in the southern hemisphere. We acknowledge that the onset and duration of ‘summer’ varies across the globe, and may not even be well-defined in some parts of the tropics; our goal was not to capture a ‘summer’ effect, but only to minimize variability in a multi-year time series by selecting a fixed time domain with low probability of freeze-thaw and a fairly stable vegetation canopy from one year to the next (see further discussion below in *Usage Notes*). To illustrate a continuous backscatter time series for these three instruments, we developed a simple intercalibration of the summer mean backscatter time series – a constant offset of the QuikSCAT backscatter PR data equal to the mean difference between summer Ku-band backscatter and summer C-band backscatter PR during the five years with overlapping data (1999–2000 for ERS and QuikSCAT, and 2007–2009 for QuikSCAT and ASCAT). We use this intercalibration to develop time series plots for visual inspection in figures below, but not for inclusion in the dataset.

### Multi-sensor backscatter at invariant non-urban sites

Large expanses of continuous tropical rainforest land cover have been extensively used to validate the calibration of ERS, QuikSCAT, and ASCAT scatterometers because of the large-scale uniformity in the backscatter response from this vegetation^36–38^. We selected four tropical evergreen forest test sites (Table 2) that we expect to have had minimal land-use/land-cover change during 1993–2020, based on location and a visible review of Google Earth image records. Sites were selected so as to exclude large water bodies or coastal grid cells. All four sites are in the southern hemisphere, so we used Jan-Feb-Mar for mean summer backscatter. We constructed a multi-sensor time series for each site by using the summer mean backscatter for each 0.05° grid cell, and then computing the aggregate mean and standard deviation for an 11 × 11 set of grid cells at each site (roughly 3500 km^2^).

**Table 2:** Evergreen tropical forest sites.

Site	Lat (°N)	Lon (°E)
Amazon1	−4.85	−62.00
Amazon2	−6.00	−66.50
DemRepCongo	−1.00	23.00
PapuaNewGuinea	−3.00	140.00

**Fig. 2:**
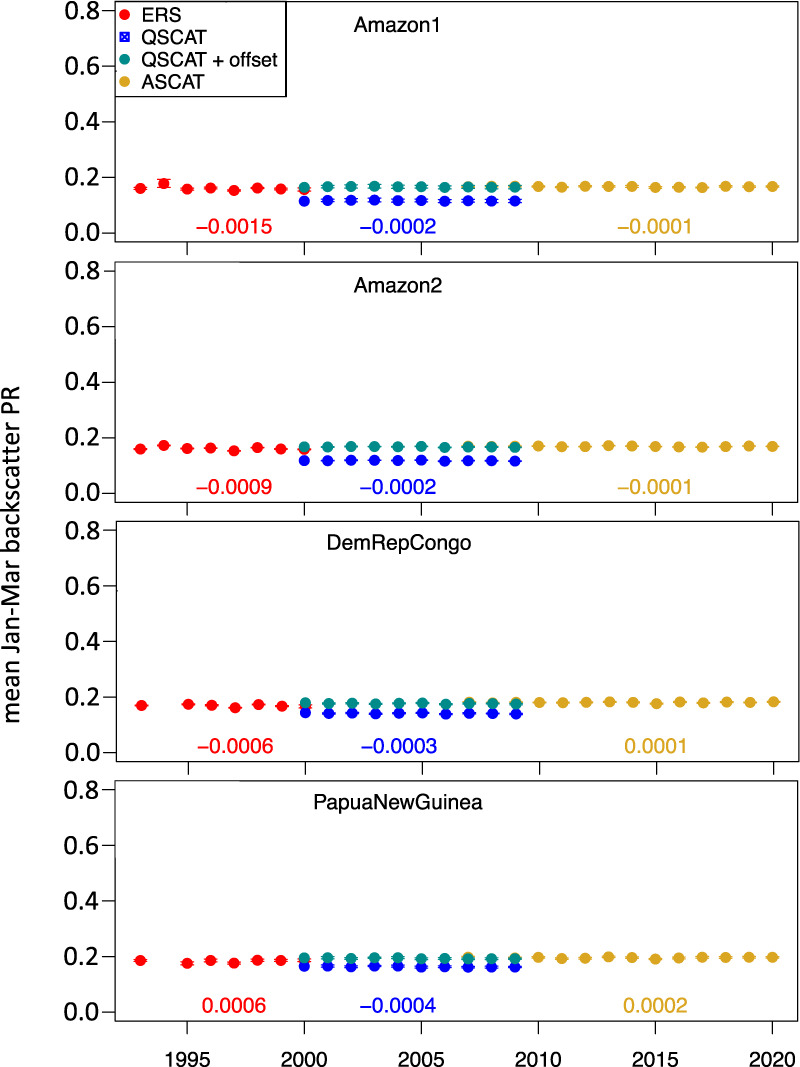
Backscatter time series for tropical evergreen forests. Mean Jan-Mar backscatter power return (PR) time series for four ‘invariant’ tropical forest sites (see Table 2 for site locations). For the QuikSCAT data, a simple Ku-band to C-band offset is computed for each site as the mean difference over the 5 overlapping years (1999–2000, 2007–2009) and QuikSCAT + offset is also plotted. Each point includes a standard deviation range for the 121 grid cells (0.05° lat/lon); in all cases this range is smaller than the symbol size. ERS backscatter data were missing for Jan-Mar 1994 at two sites. Linear fits (backscatter vs. year) are computed separately for ERS, QuikSCAT, and ASCAT data; trends (yr^−1^) are reported below the data points. Note: y-axis scale matches the y-axis scales of Figs. 3–6 below.

QuikSCAT Ku-band tropical evergreen forest backscatter PR is generally 0.02–0.05 lower than ERS and ASCAT C-band tropical evergreen forest backscatter (Fig. 2); this corresponds to roughly 0.7 dB to 1.3 dB lower σ° values, and is due primarily to the difference in sensor frequency/wavelength. For each site, we computed a simple Ku-band to C-band intercalibration offset as the mean difference between the five summer seasons with overlap (1999 & 2000 for ERS and QuikSCAT; 2007–2009 for QuikSCAT and ASCAT). The evergreen tropical forest backscatter signal is generally stable over 1993–2020, and the constant offset QuikSCAT_off_ generates a rather constant backscatter if QuikSCAT + offset is combined with ERS and ASCAT (Fig. 2). For the four sites, trends in backscatter PR for each instrument ranged from −0.0015 to +0.0006 yr^−1^ (ERS), −0.0004 to −0.0002 yr^−1^ (QuikSCAT), and −0.0001 to +0.0002 yr^−1^ (ASCAT). Only for one site and sensor (QuikSCAT, Papua New Guinea) was the linear trend significant at p < 0.05.

### Summer ASCAT backscatter compared to building data

Mathews *et al*.^16^ reported validation results comparing QuikSCAT Ku-band backscatter to lidar-derived building height for nine US cities, ranging in area (of lidar data coverage) from 70 km^2^ (Atlanta) to 8297 km^2^ (Washington DC). For each city, they had a single-year lidar snapshot of building height, and selected QuikSCAT data from that same year. QuikSCAT data were processed with the Dense Sampling Method^14^ to generate annual mean backscatter at 1-km resolution, and lidar data were aggregated to the same 1-km grid. Resulting coefficients of association strength (R^2^) ranged from 0.04 (significant at p-value < 0.05) to 0.32 (significant at p-value < 0.01). Both QuikSCAT and lidar data were then converted to spatial trends by fitting polynomial functions over each city, generating a broad, smooth oblong domed shape for each city, elliptical in plan view with major and minor axis length differences. These trended datasets were more strongly associated, with R^2^ values ranging from 0.33 to 0.98 (all significant with p-values < 0.01).

We compared 2015 summer mean ASCAT C-band backscatter to a continental-scale data set of building volume and building height^39^, which used random forest machine learning, with multiple remote sensing and other spatial input data sets (e.g., Landsat, MODIS, Sentinel-1 SAR, DEM, roads) to generate 1-km gridded data sets of c.2015 building height (m) and building volume (10^5^ m^3^ km^−2^) for urban areas across China, the USA, and Europe (Russia, Ukraine, and Belarus were not included in the building dataset). The random forest model was fit using a subset of available collections of lidar data, and tested against lidar data withheld from the fitting^39^. We used the results of the ‘combined’ model, where the models of building height, density, and volume are generated using data aggregated from all three regions in (China + USA + Europe); results for individual models of China, USA, Europe were similar^39^. These 1-km gridded building dimensional data were aggregated to 0.05° × 0.05°. We compared the building volume and height data to ASCAT Jul-Sep mean backscatter for 215 large cities (population >1 M [ref. ^2^]) in the three regions, extracting backscatter and building data for 11 × 11 grids of 0.05° grid cells for each city (roughly 2000–3500 km^2^, depending on latitude). Following Matthews *et al*.^16^, the building dimensional data were further smoothed, using a 5 × 5 grid moving mean window.

**Fig. 3:**
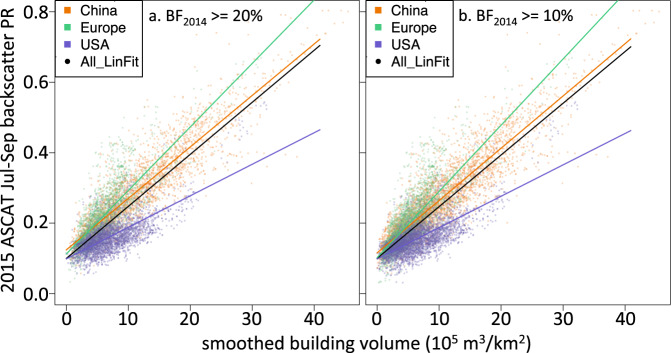
Urban backscatter vs. building volume. Mean summer 2015 ASCAT backscatter power return (PR) vs. smoothed building volume for 0.05° grid cells in 200+ major cities masked by (**a**) GHSL 2014 Built Fraction >=20%, and (**b**) GHSL 2014 Built Fraction >=10%. Data disaggregated by region: China (orange), USA (blue), Europe (green). All urban areas are in the northern hemisphere, so mean Jul-Sep backscatter is used. Linear fits for each region are shown in colored lines, linear fit for all data is the black line. Linear fit statistics are reported in Table 3, for both ASCAT PR vs. building volume, as in figures above, and ASCAT PR vs. building height (figures not shown).

Applying a minimum 2014 built fraction threshold of 20% [refs. ^2,33^], there were 8450 urban grid cells (0.05°) across the 215 cities in China, Europe, and the USA that were used for comparison with the Li *et al*.^39^ building metrics data; this increased to 11,548 grid cells with a lower built fraction threshold of 10% (Table 3). Correlations between building volume and C-band backscatter were strong (Fig. 3; Table 3), with all significant at p < 0.001. Lowering the built fraction threshold to 10% led to a small increase in the correlation coefficients. Correlations between building height and ASCAT C-band backscatter were slightly weaker (Table 3), and again all significant at p < 0.001. In general, backscatter was lower for USA urban grid cells than for urban grid cells in China or Europe with similar building volumes (Fig. 3), as were the linear fit slopes (Table 3).

**Table 3:** 2015 ASCAT summer mean urban backscatter PR correlation to 2015 building volume and 2015 building height^39^, using two GHSL BF_2014_ thresholds for the urban mask.

	GHSL BF_2014_ >= 20%				GHSL BF_2014_ >= 10%			
	China	Europe	USA	All	China	Europe	USA	All
*# grid cells*	2690	2405	3355	8450	4148	3429	3971	11548
***building volume***								
*linear fit R*^2^	0.83	0.65	0.52	0.69	0.83	0.69	0.53	0.70
*linear fit slope*	0.015	0.018	0.009	0.015	0.015	0.019	0.009	0.015
±*stand. error*	±*0.0001*	±*0.0003*	±*0.0001*	±*0.0001*	±*0.0001*	±*0.0002*	±*0.0001*	±*0.0001*
***building height***								
*linear fit R*^2^	0.76	0.56	0.32	0.65	0.76	0.58	0.34	0.65
*linear fit slope*	0.031	0.036	0.014	0.031	0.031	0.034	0.014	0.030
±*stand. error*	±*0.0003*	±*0.0007*	±*0.0003*	±*0.0002*	±*0.0003*	±*0.0005*	±*0.0003*	±*0.0002*

## Usage Notes

### Spatio-temporal data limitations

Within the overall land domain (+64.5° to −58°N, +180 to −180°E), data are missing for numerous islands, including the Hawaiian Islands USA, and small island nations, e.g., Mauritius and Reunion. Within the overall QuikSCAT temporal domain (1999–2009), Jan-Jun 1999 and Dec 2009 are not included in the monthly data files as QuikSCAT was not collecting data then, and the first two seasons of 1999, Jan-Mar/1999 and Apr-Jun/1999, have no data values, the third season, Jul-Sep/1999, is based on backscatter from July 19, 1999 through September 1999, and the final season of 2009, Oct-Dec/2009, is based on backscatter from October 2009 through Nov. 21, 2009.

The urban backscatter signal from scatterometers is affected by urban land change and densification. The grid does not resolve variations on the sub 1-km scale. As a result, the spatial domain constraints allow the data to be used on the WGS-84 standard coordinate system, as well as other coordinate systems, like GCJ-02 and BD-09, with minimal concerns.

**Fig. 4:**
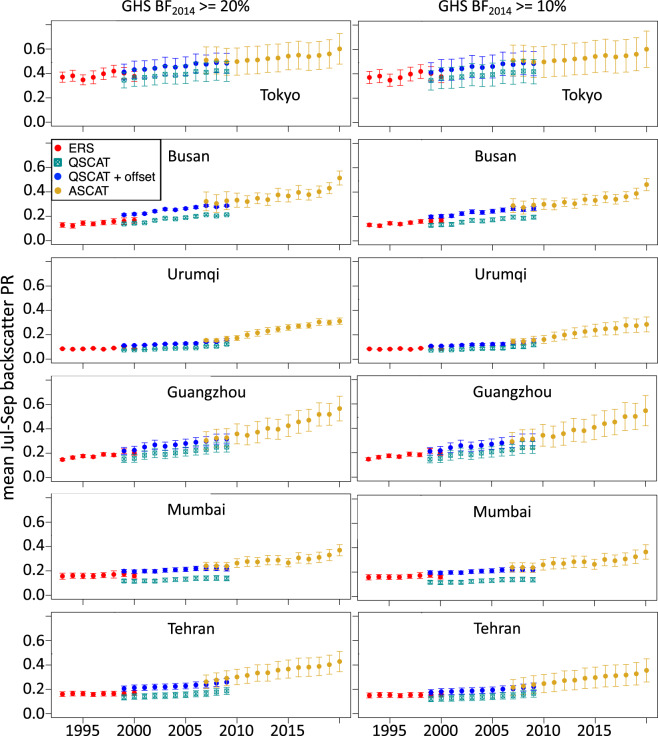
Backscatter time series for large cities. Mean summer backscatter power return (PR) time series for six large cities, using ERS, QuikSCAT, and ASCAT data. Centered on each city, an 11 × 11 grid of 0.05° lat/lon grid cells was selected, masked to remove open water >50%, and built fraction in 2014 (left column) <20%, or (right column) <10%. Mean and standard deviations of remaining grid cells are plotted. For the QuikSCAT data, a simple Ku-band to C-band offset is computed for each site as the mean difference over the 5 overlapping years (1999–2000, 2007–2009) and QuikSCAT + offset is also plotted.

**Fig. 5:**
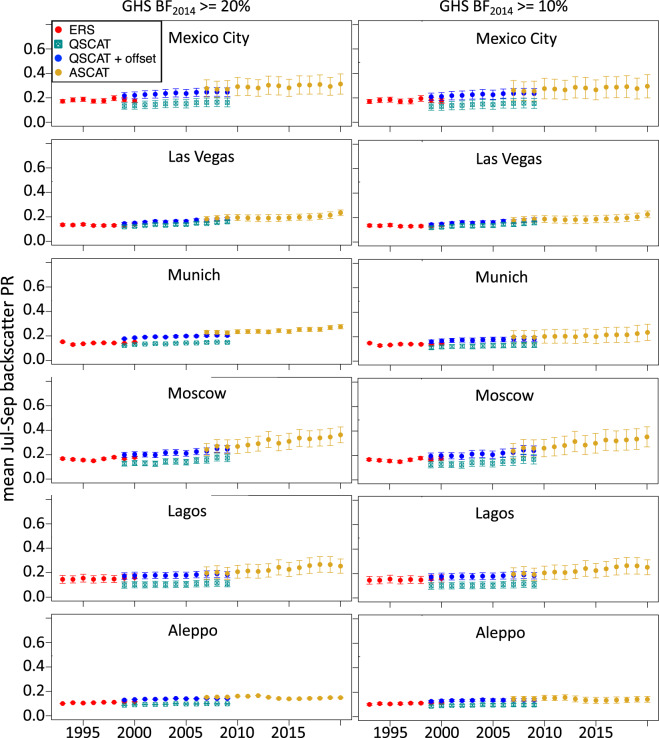
Backscatter time series for large cities. Mean summer backscatter power return (PR) time series for six large cities, using ERS, QuikSCAT, and ASCAT data. Centered on each city, an 11 × 11 grid of 0.05° lat/lon grid cells was selected, masked to remove open water >50%, and built fraction in 2014 (left column) <20%, or (right column) <10%. Mean and standard deviations of remaining grid cells are plotted. For the QuikSCAT data, a simple Ku-band to C-band offset is computed for each site as the mean difference over the 5 overlapping years (1999–2000, 2007–2009) and QuikSCAT + offset is also plotted.

This urban backscatter data set has been developed to investigate multi-decadal trends in urban growth, which for most cities over 1993–2020 has been essentially monotonically increasing. In order to filter out short-term variability in urban backscatter due to factors like vegetation seasonality, precipitation and surface wetting, freeze-thaw^15^, we have developed a three-month mean backscatter time series data set, and would recommend using one specific three-month period, e.g., the summer period, to construct an annual time series of change, as presented in Figs. 2, 4 and 5, and as used to compare to building volume (Fig. 3, Table 3) and building height (Table 3). To restrict the data to urban land, in the *Seasonal_Urban_sig0_dB* dataset, we excluded grid cells with <20% built fraction in 2014. As shown in Figs. 3–5 and Table 3, using a less restrictive built-fraction mask of <10% generated similar results. Using the 20% built fraction threshold on the GHSL coverage for 2000 or 1990 would have included 14% or 24% fewer grid cells globally, respectively. In our city-scale summary of the data below we selected urban grid cells within an 11 × 11 grid (0.05° cells, so roughly 50 km x 50 km, depending on latitude) centered on large cities, to minimize the influence of sub-urban and rural land outside the cities.

To allow users to experiment with different urban masks, we also have generated an unmasked land data set for each sensor (e.g., *{sensor}_seasonal_Land_sig0_mean.nc*). To allow users to explore different questions about urban backscatter seasonality with different temporal aggregations, we also have generated a monthly unmasked land data set for each sensor (e.g., *{sensor}_monthly_land_sig0_mean.nc*).

There is a difference in magnitude between C-band vs. Ku-band urban backscatter. We computed offsets for invariant tropical evergreen forest sites (Fig. 2) and major cities (e.g., Figs. 4 & 5 below); the offset magnitude varied from site to site, and no clear pattern was evident when this city-scale offset was mapped globally. However, as both the invariant sites and example cities show, a simple local offset can be computed that generates a fairly consistent overlapping 28-year time series. Inter-sensor differences are more likely due to urban backscatter differences caused by sensor geometry and wavelength differences than to changes in the urban structure from decade to decade. We do not consider the offset QuikSCAT backscatter that we computed to be a data product, and use it only to better illustrate the data. We do not recommend combining ERS and QuikSCAT_offset_ and ASCAT into a single variable time series until a more rigorous intercalibration is done; instead, we recommend that each sensor’s time series should be evaluated independently, though trends across the three sensors/eras should be intercomparable. We also do not recommend quantitative intercomparison between particular cities until more research is done on the impact of urban surface properties on backscatter strength; instead, we recommend evaluating temporal trends in backscatter for individual large cities or aggregated to large regions.

Several other scatterometers have flown over the past few decades, but were not appropriate to include in this data set due to shorter time intervals or data availability. For example, SeaSAT (6/1978 – 10/1978), NSCAT (8/1996 – 6/1997), SeaWinds on ADEOS-2 (3/2003–10/2003), and Rapidscat (10/2014–8/2016) all have short data records. The Indian Ku-band scatterometers OSCAT-1 (10/2009–2/2014) and OSCAT-2 on SCATSat-1 (8/2016–3/2021) together have a quasi-decadal record, but examination of the OSCAT-1 data showed enough striping and data gaps that it was not included in this analysis. Chinese scatterometers (e.g., the Ku-band HY series) have been operating in the 2010s, but these data have not been made available to the NASA SCP for processing.

### Urban applications

Highly-built urban landscapes have much higher backscatter than evergreen tropical forests – compare Fig. 2 to Figs. 4 & 5 below – despite evergreen broadleaf forests having higher backscatter than other vegetated land cover types^40^. In addition, the change in urban backscatter over a decade, particularly QuikSCAT in the 2000s and ASCAT in the 2010s (Figs. 4 & 5), can be much larger than variation or trends in evergreen tropical forests (Fig. 2), indicating that urban trends are not due to instrument drift, but rather to changes in urban surface characteristics.

A 10-m resolution model of building height in German cities, using Sentinel-1 SAR data, under-predicts the number of buildings with heights >20–30 m, and appears to have a saturation effect at about 20 m [ref. ^13^]. The relationship of scatterometer backscatter to building volume doesn’t seem to saturate, even for mega-cities in China (Fig. 2). This lack of saturation in the backscatter data may be due to a spatial averaging effect on the complicated nature of microwave backscatter from the built environment^12^, when reconstructing signals at ~10-km spatial resolution (urban districts) vs. 10-m resolution (individual buildings). We found a stronger linear correlation between 2015 ASCAT backscatter and building volume (Fig. 3) when backscatter values were in power return ratio, PR = 10^(σ^0^/10), where σ^0^ is the backscatter in dB, than the relationship between building volume and backscatter σ^0^ in dB. The data sets are posted as backscatter σ^0^ in dB, but our analysis figures and tables use backscatter PR.

As an example of data usage, time series of summer mean urban backscatter 1993–2020, aggregated to individual large cities were generated for 12 large cities, and show a range of trends in urban growth at the city scale. For each city, an 11 × 11 grid of 0.05° lat/lon grid cells was selected, masked to remove grid cells with open water fraction >50% and/or built fraction in 2014 <20% (or <10%, for comparison) and a city-wide summer mean backscatter was computed for each year and sensor (Figs. 4 & 5). To reduce the influence of sub-urban and rural land outside the city and to be consistent with our earlier work^2^,^18^ we restricted the domain of each city to an 11 × 11 grid. We note that neither the urban nor the all-land data sets posted at SEDAC are masked by distance from a city center, so different urban extents can be evaluated in future research.

The lower BF threshold (10%) includes a higher number of grid cells in almost all cases (Figs. 4 & 5). Differences between the two sets of panels in Figs. 4 and 5 (BF_min_ = 20% or BF_min_ = 10%) are small, though the lower threshold generally has slightly lower means and slightly higher variance across a city. There are clear differences between cities in the rates and timing of urban backscatter change over 1993–2020, although all of the example cities show mostly monotonic increase except Aleppo, Syria (Fig. 5), where backscatter dropped from 2012–2014 and had not recovered by 2020, consistent with a drop in night time lights in Aleppo from 2012–2015 resulting from the Syrian Civil War^41^.

The validation and urban time series analyses presented here were restricted to 11 × 11 blocks of the 0.05° × 0.05° grids (~2000–3500 km^2^), centered on large cities (population >1 M), following Mahtta *et al*.^2^ Microwave backscatter resolution is relatively coarse, though signal processing can sacrifice temporal resolution to improve spatial resolution^14,30^. We have targeted data for quantifying multi-decadal, global urban change at the scale of large cities (e.g., Figs. 4 & 5) to large regions. We caution against using these data sets to address questions about small cities (one to a few 0.05° grid cells, scale ~ 100 km^2^), or detailed analysis of spatial patterns within a single city.

Urban backscatter will be affected by a number of structural and architectural factors that vary across the world’s cities – for example, building materials, angular vs. rounded architecture, and the urban road network layout (gridded vs. ‘random’^15^). Sufficient granular data (i.e., global at less than 5–10 km resolution) are not available to resolve the impact of these effects. This variation will contribute to the scatter shown in Fig. 3, and makes it difficult to conduct detailed intercomparisons between cities. Until this variability is better studied and understood, we recommend that these urban backscatter data be used for regional-scale analyses, assessments of individual cities over time, and conclusions be confined to general patterns.

**Fig. 6:**
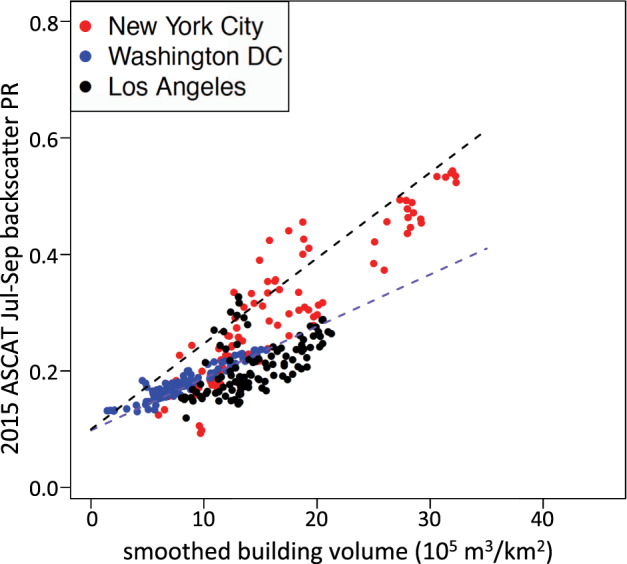
Urban backscatter vs. building volume for New York City, Los Angeles, and Washington DC. Mean summer 2015 ASCAT backscatter power return (PR) vs. smoothed building volume^40^, using the GHS BF_2014_ ≤0.2 mask. Note: panel axis scales match Fig. 3; black (blue) dashed line is linear fit to all regions (USA) data from Fig. 3.

The overall relationship between ASCAT backscatter and building volume was quite different for the USA (56 large cities) than for China (113 large cities) or Europe (48 large cities), with USA cities having a much lower increase in backscatter per increase in building volume (Fig. 3). Comparison of backscatter to building height was similarly different for the USA (Table 3). Understanding what this difference implies about the urban built environment is an open research question. Looking at a few individual cities in the USA (Fig. 6), the backscatter-volume relationship for New York City is similar to the fit to all regions (see Fig. 3a above), while the relationships for Los Angeles and Washington DC are more representative of the average USA relationship. Some inter-city differences in backscatter may be due to differences in the surrounding environment, e.g., built sprawling suburbs, un-built arid or mountainous or forested or cropped land, or open water. The magnitude of these effects will depend on the degree of backscatter signal contribution from the surrounding environment to the selected urban grid cells.

The combination of high resolution sensors and increasing capabilities to process large data sets should lead to higher resolution global urban structure data sets than these scatterometer data provide, for example, the SAR-based map of building height at 10-m resolution for Germany^13^. Since SAR data from Sentinel-1 is not available for the 1990s or 2000s, a multi-decadal building height time series from these SAR data cannot be developed until another decade of data are collected. Scatterometer data can provide an important prequel to these new SAR-based building data being developed, with global coverage for several decades of rapid urban growth, though further research would be needed to understand their relationships. This scatterometer data set will remain an important global urban built structure data set for the 1990–2020 era, a period of very rapid urban growth.

## Data Availability

Code used in this paper is available in the following locations: The github repository, tmilliman/sir_to_netcdf, (https://github.com/tmilliman/sir_to_netcdf) includes the python and bash scripts that: 1. Convert NASA SCP backscatter SIR images to NetCDF files of seasonal mean and standard deviation backscatter at 0.05° (lat/lon) for all land, and with an urban mask. Separate code for each sensor (ERS, QuikSCAT, ASCAT). A separate github repository, tmilliman/urban_backscatter, (https://github.com/tmilliman/urban_backscatter) includes scripts that: 2. Extract from NetCDF files in #1 the backscatter data for 11 × 11 grids around a lat-lon location and create CSV files from this data. 3. Create using the scripts in #2 CSV files with the mean backscatter for city-level grids around city centers and for also for the invariant regions shown in Fig. 3. The github repository sfrolking/urban_backscatter_ERS_QSCAT_ASCAT (https://github.com/sfrolking/urban_backscatter_ERS_QSCAT_ASCAT) includes R scripts that: 4. Evaluate seasonal backscatter for invariant evergreen tropical forest sites and construct Fig. 2. 5. use the CSV files from #3 above and correlate 2015 summer mean ASCAT backscatter with building volume data and construct Fig. 3. 6. use the CSV files from #3 above to generate annual mean summer backscatter time series plots for sample cities and construct Figs. 4–6.
